# Blood–Brain Barrier Leakage during Early Epileptogenesis Is Associated with Rapid Remodeling of the Neurovascular Unit

**DOI:** 10.1523/ENEURO.0123-18.2018

**Published:** 2018-05-30

**Authors:** Marion Bankstahl, Heike Breuer, Ina Leiter, Martin Märkel, Pablo Bascuñana, Dominik Michalski, Frank M. Bengel, Wolfgang Löscher, Martin Meier, Jens P. Bankstahl, Wolfgang Härtig

**Affiliations:** 1 Department of Pharmacology, Toxicology and Pharmacy, University of Veterinary Medicine Hannover and Center for Systems Neuroscience, Bünteweg 17, Hannover, 30559, Germany; 2 Department of Nuclear Medicine, Hannover Medical School, Hannover, 30625, Germany; 3 Paul Flechsig Institute for Brain Research, University of Leipzig, Liebigstr. 19, Leipzig, 04103, Germany; 4 Department of Neurology, University of Leipzig, Liebigstr. 20, Leipzig, 04103, Germany; 5 Preclinical Imaging Labs, Central Laboratory Animal Facility & Institute for Laboratory Animal Science, Hannover Medical School, Hannover, 30625, Germany

**Keywords:** Blood–Brain Barrier, Epileptogenesis, MR Imaging, Neurovascular Unit

## Abstract

Increased permeability of the blood–brain barrier (BBB) following cerebral injury results in regional extravasation of plasma proteins and can critically contribute to the pathogenesis of epilepsy. Here, we comprehensively explore the spatiotemporal evolution of a main extravasation component, albumin, and illuminate associated responses of the neurovascular unit (NVU) contributing to early epileptogenic neuropathology. We applied translational *in vivo* MR imaging and complementary immunohistochemical analyses in the widely used rat pilocarpine post–status epilepticus (SE) model. The observed rapid BBB leakage affected major epileptogenesis-associated brain regions, peaked between 1 and 2 d post-SE, and rapidly declined thereafter, accompanied by cerebral edema generally following the same time course. At peak of BBB leakage, serum albumin colocalized with NVU constituents, such as vascular components, neurons, and brain immune cells. Surprisingly, astroglial markers did not colocalize with albumin, and aquaporin-4 (AQP4) was clearly reduced in areas of leaky BBB, indicating a severe disturbance of astrocyte-mediated endothelial-neuronal coupling. In addition, a distinct adaptive reorganization process of the NVU vasculature apparently takes place at sites of albumin presence, substantiated by reduced immunoreactivity of endothelial and changes in vascular basement membrane markers. Taken together, degenerative events at the level of the NVU, affecting vessels, astrocytes, and neurons, seem to outweigh reconstructive processes. Considering the rapidly occurring BBB leakage and subsequent impairment of the NVU, our data support the necessity of a prompt BBB-restoring treatment as one component of rational therapeutic intervention to prevent epileptogenesis and the development of other detrimental sequelae of SE.

## Significance Statement

Blood–brain barrier (BBB) leakage is critically involved in brain insult–mediated epilepsy development. Here, we demonstrate rapid but transient BBB damage within hours after experimental status epilepticus (SE), an epileptogenic insult, and subsequent degenerative events at the level of the so-called neurovascular unit (NVU), which reflects the anatomic and functional interplay between brain vasculature, glial cells, and neurons. Analyses at cellular level revealed degeneration of various NVU components, which seem to outweigh reconstructive processes, thus providing potential targets for protective pharmacotherapy. The findings emphasize the requirement and expedience of rapid BBB-stabilizing treatment as a primary element of epilepsy-preventive therapeutic interventions.

## Introduction

Different kinds of cerebral injury, such as head trauma, stroke, cerebral infection, and status epilepticus (SE), may lead to the development of epilepsy in a certain proportion of affected patients (Pitkänen and Immonen, 2014; Schmidt and Sillanpää, 2016). The mechanisms subjacent to the process of epileptogenesis following brain insults, though, are not well understood. One common characteristic of epileptogenic brain insults is impairment of the blood–brain barrier (BBB). A leakage of the BBB results in extravasation of albumin and subsequent activation of glia and inflammatory responses and is considered one likely key factor triggering epileptogenesis (Seiffert et al., 2004; Ivens et al., 2007; van Vliet et al., 2007; Marcon et al., 2009).

Albumin extravasation into the brain, eventually resulting in recurrent electrographic seizures (Bar-Klein et al., 2014; Weissberg et al., 2015), is observed in various post-SE models of epileptogenesis (van Vliet et al., 2007; Michalak et al., 2013; Breuer et al., 2017). Importantly, increased BBB permeability is currently under investigation as a potential prognostic biomarker for stratifying individuals at risk to develop epilepsy following epileptogenic brain insults (Pitkänen et al., 2016; Walker et al., 2016; Bar-Klein et al., 2017) and as a treatment target for attenuation or prevention of epileptogenesis by applying drugs stabilizing or restoring BBB function during the latency phase preceding appearance of the first clinical seizure (Friedman and Heinemann, 2012; Janigro, 2012; van Vliet et al., 2016). For both objectives, it is crucial to reveal the *in vivo* spatiotemporal pattern of BBB leakage following cerebral injury. Furthermore, elucidating cellular and parenchymal distribution patterns of extravasated serum albumin and associated changes in the neurovascular unit (NVU) will be beneficial for better understanding of pathologic cascades finally leading to chronic seizure generation. In view of the broad application of the pilocarpine rat model, which is one of the most widely used rodent models of epileptogenesis and has brought a large body of information relevant to epilepsy development and its prevention, we consider it important to further characterize this model. Moreover, recent studies in a refined version of this model (Bröer and Löscher, 2015; Brandt et al., 2015) argue for its beneficial application for predictive biomarker identification. Further, severe SE itself is a common and life-threatening neurologic condition. Understanding its consequences is of high importance, also considering its induction by cholinergic nerve agents released by chemical weapons (Tang et al., 2011).

Therefore, we first present detailed data on the *in vivo* spatiotemporal pattern of BBB leakage during epileptogenesis in the lithium-pilocarpine post-SE model of epileptogenesis in rats assessed by contrast-enhanced MR imaging for which we recently published a methodological paper identifying the most suitable translational imaging approach (Breuer et al., 2017). Second, the cellular uptake and extracellular distribution pattern of fluorescein-linked albumin were determined. Third, multiple fluorescence staining was applied to identify colocalization of extravasated serum albumin with various histochemical markers for cellular and acellular constituents of the extended NVU, i.e., BBB endothelium, the vascular basement membranes, the glia-endothelial interface, astrocytes, microglia, and neurons.

## Materials and Methods

### Animals

Adult female Sprague-Dawley rats (200–220 g, *n* = 60) were obtained from Harlan Laboratories. They were housed in pairs under controlled climate conditions (22 ± 1°C, humidity 45%–55%) in individually ventilated cages under a 14/10-h light-dark cycle (rats used for imaging experiments) or in groups of 5 in open cages (22 ± 1°C, humidity 45%–55%) under a 12/12-h light-dark cycle (rats used for immunohistochemistry). Standard diet (Altromin 1324; Altromin) and water were accessible *ad libitum*. After delivery, animals were allowed to adapt to the new conditions, repetitively handled for at least 1 wk before being subjected to experiments, and randomized to experimental groups. Experiments were conducted in accordance with European Communities Council Directives 86/609/EEC and 2010/63/EU and were formally approved by the responsible local authority.

### Chemicals, drugs, and antibodies

Isoflurane (Isofluran Baxter) was obtained from Baxter and CP-Pharma, diazepam as commercial solution (Faustan or Diazepam-ratiopharm) from Temmler Pharma or Ratiopharm, respectively, and glucose electrolyte solution (Sterofundin HEG-5) from B. Braun (Melsungen, Germany). Gadolinium-DTPA (Gd-DTPA, Magnevist 0.5 mmol/ml) was purchased from Bayer HealthCare. All immunoreagents were obtained from Dianova as supplier for Jackson ImmunoResearch. Unless stated otherwise, all further chemicals were of analytic grade and purchased from Sigma-Aldrich.

### Induction of status epilepticus

SE was induced in rats (*n* = 42) as described elsewhere (Bankstahl et al., 2012). In brief, 14–16 h after the administration of lithium chloride (127 mg/kg in 3 ml/kg 0.9% saline, p.o.) and 30 min after methyl scopolamine (1 mg/kg in 2 ml/kg 0.9% saline, i.p.), injection of pilocarpine (10 mg/kg, repeated up to 5 times, in 1 ml/kg 0.9% saline, i.p.) was repeated until SE, which was characterized by the onset of repetitive generalized convulsive seizures (stage 4 or 5) without intermediate recovery of normal behavior. SE was interrupted after 90 min by administration of diazepam (10 mg/kg in 2 ml/kg, i.p.). Diazepam injection was repeated after 15 min (10 mg/kg), and, if needed, after 30 min using half of the first dose (5 mg/kg). Self-sustaining SE was successfully established in 90.5% of animals, which required an average pilocarpine dose of 35.4 ± 9.6 mg/kg (mean ± SD). Three rats in which SE could not be induced served as an additional control group for albumin extravasation. The overall mortality rate was zero. Age-matched control rats (*n* = 10) for histologic analysis were treated identically but received saline instead of pilocarpine. After SE, rats were hand-fed with mashed laboratory chow and received injections of glucose-electrolyte solution until they resumed normal feeding behavior.

### Magnetic resonance imaging

Rats were scanned before (baseline, *n* = 13) and 48 h (*n* = 5), 4 d (*n* = 6), and 10 d (*n* = 5) following SE. MRI was conducted as described recently (Breuer et al., 2017) on a 7-T (300-MHz) small animal MRI system (Bruker Pharmascan) using ParaVision 5.1 acquisition software (Bruker). Rats were anesthetized with isoflurane, and a catheter was placed in a lateral tail vein for contrast agent infusion. Following transfer of rats into the imaging chamber, which was constantly kept at 37°C, the rat maxilla was placed in a tooth bar for comparable positioning. The receive coil was placed at a defined position over the rat head. Breathing rate of rats during image acquisition was kept at 40–60 breaths/min. T2-weighted 2D multislice-multiecho (MSME) images (repetition time, 2500 ms; echo time, 11 ms; 96 slices of 0.8 mm; 256 × 256 matrix; 35 × 35 ×-25.6 mm^3^ field of view) were acquired for detection of brain edema. T1-weighted images were acquired using a 3D modified driven equilibrium Fourier transform method (MDEFT; 0.8 mm slice thickness, 256 × 256 × 32 matrix, 35 × 35 × 25.6-mm^3^ field of view) before and 30 min after start of contrast agent infusion. The resulting voxel size was 0.136 × 0.136 × 0.8 mm^3^. Gd-DTPA was intravenously infused via a syringe pump (PHD Ultra, Harvard Apparatus) using a 20-min step-down infusion schedule as described recently (Breuer et al., 2017).

### Image analysis

MRI data were coregistered to a rat brain atlas published by Schwarz et al. (2006) using PMOD software (PMOD Technologies), and data from six brain regions (hippocampus, thalamus, amygdala, piriform cortex, entorhinal cortex, and cerebellum) were extracted. T1 and T2 signals were normalized to pons, for which no alterations in contrast agent uptake or T2 MRI signal was observed after SE (Breuer et al., 2017). Voxelwise comparison to baseline (two-sample unpaired *t* test; significance level threshold, 0.001; minimum cluster size, 100 voxels) led to Gd-DTPA leakage *t*-maps using Matlab software (MathWorks) and SPM12 (UCL) as described earlier (Breuer et al., 2017).

### FITC-albumin infusion and brain slicing

For histologic analysis of albumin extravasation, rats were infused with 100 mg/kg bovine albumin–fluorescein isothiocyanate conjugate (FITC-albumin) diluted in 10 ml/kg of 0.1 m PBS at a rate of 1 ml/min under short isoflurane anesthesia before SE (control, *n* = 10) and 5 h (*n* = 6), 24 h (*n* = 5), or 48 h following SE (*n* = 16). Two hours later, rats were perfused with 125 ml of 0.01 m PBS followed by 250 ml of 4% paraformaldehyde in 0.1 m PBS at a flow rate of 16.6 ml/min. Following removal, brains were kept for 24 h in 4% phosphate-buffered paraformaldehyde for postfixation and were then stored in 30% sucrose in 0.1 m PBS with 0.2% sodium azide at 4°C until sectioning. Coronal sectioning of the forebrain of each rat was performed using a freezing microtome (Frigomobil 1205; Jung) and a slice thickness of 30 µm. For analysis of FITC-albumin extravasation patterns, series comprising each 10th coronal serial section from the forebrains of all rats were washed 3 times with TBS for at least 10 min, briefly rinsed with distilled water, mounted onto fluorescence-free slides (Menzel), air-dried, and coverslipped with Entellan (Merck).

### Semiquantitative analysis of extra- and intracellular FITC-albumin

The extent of extracellular and cellular FITC-albumin uptake was scored blinded to experimental groups. Whole-brain slice images were acquired using an all-in-one fluorescence microscope (Biorevo, BZ-9000E, Keyence). Four coronal brain sections were analyzed per animal (–0.36, –1.56, –3.72, and –4.9 mm relative to bregma; Paxinos and Watson, 2007). Target regions were hippocampus, thalamus, amygdala, piriform cortex, entorhinal cortex, and caudate putamen. Scoring was performed separately for cellular and extracellular FITC-albumin occurrence: (A) cellular FITC-albumin uptake: score 0, no cellular uptake; score 1, sporadic cellular uptake; score 2, medium amount of labeled cells, brain region is only affected in parts; and score 3, high amount of labeled cells, brain region is globally affected; and (B) extracellular FITC-albumin uptake: score 0, no extracellular FITC-albumin; score 1, minimal extracellular uptake, often near to blood vessels; score 2, medium extracellular FITC-albumin accumulation, rather focal; and score 3, high-grade extracellular FITC-albumin accumulation, rather global. Target regions were scored in both hemispheres, and the means of left and right and of the four section levels were calculated.

### Quantification of albumin extravasation following conversion into a light-microscopically visible adduct

The immunohistochemical conversion of the FITC-albumin signal into a light-microscopically visible adduct based on an anti–fluorescein-horseradish peroxidase (HRP) conjugate (Dianova) and nickel-enhanced diaminobenzidine (DAB-Ni) as chromogen for the marker enzyme. For this conversion, serial sections were extensively rinsed with TBS followed by abolishing of endogenous peroxidase activity within the tissue by treatment with 0.6% hydrogen peroxide in TBS for 30 min. After 3 further rinses with TBS for 10 min each, nonspecific binding sites for the immunoreagent were blocked with TBS containing 2% bovine serum albumin and 0.3% Triton X-100 (TBS-BSA-T) for 30 min. Subsequently, all sections were incubated with anti–fluorescein-HRP (1:2000 in TBS-BSA-T) for 2 h. Next, the sections were rinsed twice with TBS and once with 0.05 m Tris buffer, pH 8, for 10 min each. The sections were then stained by reacting for 4 min with a DAB-Ni solution [containing 40 mg nickel ammonium sulfate, 2 mg DAB tetrahydrochloride, and 5 µl hydrogen peroxide (30%) in 10 ml of 0.05 m Tris buffer, pH 8].

For quantification of FITC-albumin extravasation, images of whole-brain sections were acquired at –0.36, –1.56, –3.72, and –4.90 mm relative to bregma by a Keyence microscope and analyzed with an image quantification software (Volocity 4.3.2, PerkinElmer). Stained areas were calculated by the total number of pixels in the stained area relative to the total area of the respective brain section as described earlier (Michalski et al., 2010).

### Multiple fluorescence staining

Furthermore, selected FITC-albumin–prelabeled sections were applied to double fluorescence staining. For this purpose, the sections were extensively rinsed with TBS before blocking of unspecific binding sites with TBS containing 5% normal donkey serum and 0.3% Triton X-100 (TBS-NDS-T). The sections were then applied to mixtures of antibodies or of antibodies and lectins as listed in Table 1. Thereby, all markers were diluted in TBS-NDS-T, and in general, the incubation time was 20 h at room temperature. Three rinses with TBS for 10 min each were followed by incubation with cocktails of carbocyanine (Cy)3- and Cy5-conjugated immunoreagents, which were used at 20 µg/ml TBS-BSA for 1 h. The omission of primary antibodies and lectins in histologic control experiments resulted in the expected absence of any cellular fluorescence labeling. Pictures at lower magnifications for Fig. 2*A*–*D* were acquired with a Keyence microscope BZ 9000, and all other pictures with a confocal laser scanning microscope LSM 510 Meta from Zeiss.

**Table 1.** Double fluorescence staining of FITC-albumin prelabeled rat forebrain tissue sections

First primary antibodies	First visualizing immunoreagents	Second primary antibodies	Second visualizing immunoreagents
Biotinylated mouse anti-NeuN (1:100; Merck Millipore)	Cy3-streptavidin	Rabbit anti-laminin (1:200; Dakocytomation)	Cy5 donkey anti-rabbit IgG
Rabbit anti-laminin (1:400; Dakocytomation)	Cy3-donkey anti-rabbit IgG	Guinea pig anti-GFAP (1:200; Synaptic Systems)	Cy5 donkey anti-guinea pig IgG
Biotinylated STL (10 µg/ml; Vector)	Cy3-streptavidin	Guinea pig anti-GFAP (1:200; Synaptic Systems)	Cy5 donkey anti-guinea pig IgG
Rabbit anti-collagen IV (1:100; Merck Millipore)	Cy3-donkey anti-rabbit IgG	Biotinylated STL (20 µg/ml; Vector)	Cy5-streptavidin
Rabbit anti-AQP4 (1:100; Alomone)	Cy3-donkey anti-rabbit IgG	Biotinylated STL (20 µg/ml; Vector)	Cy5-streptavidin
Rabbit anti-AQP4 (1:100; Alomone)	Cy3-donkey anti-rabbit IgG	Guinea pig anti-GFAP (1:200; Synaptic Systems)	Cy5 donkey anti-guinea pig IgG
Rabbit anti-S100β (1:600; Synaptic Systems)	Cy3-donkey anti-rabbit IgG	Guinea pig anti-GFAP (1:200; Synaptic Systems)	Cy5 donkey anti-guinea pig IgG
Rabbit anti-Iba (1:400; Synaptic Systems)	Cy3-donkey anti-rabbit IgG	Guinea pig anti-GFAP (1:200; Synaptic Systems	Cy5 donkey anti-guinea pig IgG
Rabbit anti-Iba (1:200; Synaptic Systems)	Cy3-donkey anti-rabbit IgG	Biotinylated STL (20 µg/ml; Vector)	Cy5-streptavidin
Biotinylated mouse anti-CD45c (1:20; Serotec)	Cy3-streptavidin	Rabbit anti-Iba (1:200; Synaptic Systems)	Cy5 donkey anti-rabbit IgG
Biotinylated mouse anti-CD8b (1:25; Serotec)	Cy3-streptavidin	Rabbit anti-Iba (1:200; Synaptic Systems)	Cy5 donkey anti-rabbit IgG

All fluorescent immunoreagents were obtained from Dianova and used at 20 µg/ml for 1 h. AQP4, aquaporin-4; GFAP, glial fibrillary acidic protein; Iba, ionized calcium binding adapter molecule 1; NeuN, neuronal nuclei; STL, *Solanum tuberosum* agglutinin (potato lectin).

### Assessment of neurodegeneration

Assessment of neurodegeneration was performed in Nissl-stained coronal brain sections in a blinded fashion with respect to the time point following SE. First, severity of neuronal damage was semiquantitatively assessed by a grading system as previously described (Bankstahl et al., 2012): score 0, no obvious damage; score 1, slightly abnormal appearance of the structure without clear evidence of visible neuronal loss; score 2, lesions involving 20%–50% of neurons; and score 3, lesions involving >50% of neurons. Scoring was performed in hippocampal subregions (CA1, CA3a), amygdala, and piriform and entorhinal cortex in four sections per rat (−2.4, −3.36, –4.68, and −5.64 mm relative to bregma according to Paxinos and Watson [2007]). Averaged scores from these sections of both hemispheres in each rat were used for calculation of group data. Second, the amount of polymorph neurons in the dentate hilus was quantified as described earlier (Polascheck et al., 2010) using AxioVision software (Zeiss). The dentate hilus was defined as the inner border of the granule cell layer and two straight lines connecting the tips of the granule cell layer and the proximal end of the CA3c region. Only cells of neuronal morphology and a diameter larger than 8 μm were counted. Per rat, three sections (at −2.4, −3.36, and −4.68 mm relative to bregma) were analyzed, and numbers of neurons were averaged from these sections.

### Statistical analysis

Statistical analyses were performed using Prism 7 software (GraphPad Software). Depending on whether data were normally distributed, either parametric or nonparametric tests were used for statistical evaluation. All rank or score data were analyzed by nonparametric tests. MRI data and data resulting from quantitative histologic analysis were analyzed by one-way ANOVA, followed by Dunnett’s multiple-comparison test comparing baseline to each time point after SE. Nonparametric data resulting from semiquantitative analysis of immunohistochemical staining were analyzed by Kruskal–Wallis ANOVA, followed by Dunn’s *post hoc* test comparing the control group with the SE groups at each time point. All tests were used two-sided. If not stated otherwise, values of *p* < 0.05 were considered statistically significant.

## Results

### *In vivo* spatiotemporal pattern of blood–brain barrier leakage during early epileptogenesis

To determine the time course of BBB impairment during epileptogenesis, rats were scanned before and 5 h, 48 h, 4 d, and 10 d post-SE using recently published contrast-enhanced T1-weighted as well as T2-weighted MRI sequences (Breuer et al., 2017).

**Figure 1.**
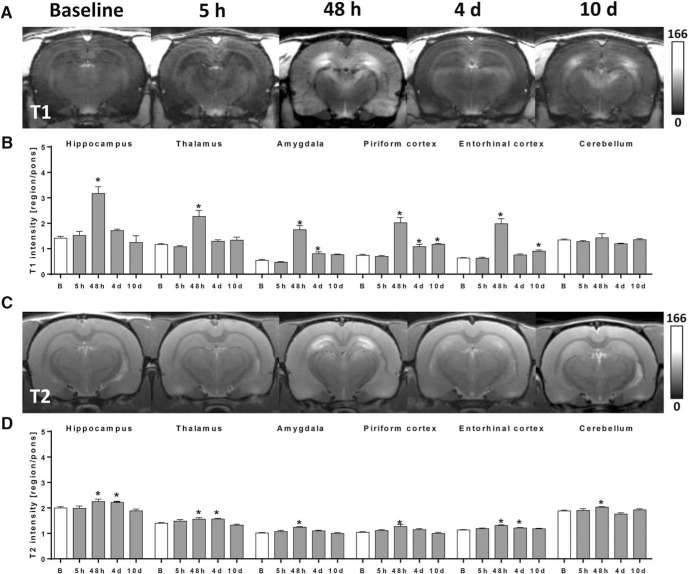
Spatiotemporal course of blood–brain barrier (BBB) impairment and cerebral edema following status epilepticus (SE) as assessed *in vivo* by 7T MRI. ***A***, Exemplary contrast-enhanced T1-weighted coronal brain images in identical gray scale displaying region-dependent severity of BBB leakage during epileptogenesis at 5 h, 48 h, 4 d, and 10 d post-SE. ***B***, Quantified T1-modified driven equilibrium Fourier transform (MDEFT) values measured after infusion of gadolinium-diethylenetriamine pentaacetic acid (Gd-DTPA) as surrogate marker for BBB leakage before (*n* = 13) and 5 h (*n* = 5), 48 h (*n* = 5), 4 d (*n* = 6), and 10 d (*n* = 5) post-SE. ***C***, Exemplary T2-weighted coronal brain images displaying region-dependent severity of cerebral edema during epileptogenesis. ***D***, Quantified T2 multislice-multiecho (MSME) values measured before (*n* = 13) and 5 h (*n* = 4), 48 h (*n* = 5), 4 d (*n* = 6), and 10 d (*n* = 5) post-SE. Data in ***B*** and ***D*** are normalized to pons and illustrated as mean ± SEM. * *p* < 0.05 versus baseline (***B***), one-way ANOVA, Dunnett’s *post hoc* test.

Gd-DTPA extravasation, resulting in elevated T1 values and indicating BBB leakage, was absent at baseline and 5 h post-SE in all investigated brain regions (Fig. 1*A*,*B*). Strongly increased T1 values versus baseline were present 48 h post-SE in epileptogenesis-associated brain regions (*p* < 0.0001 for all brain regions), but not in the cerebellum (Fig. 1*B*). The maximal signal enhancement occurred in the amygdala, with a T1 intensity increase of 221%. At 4 and 10 d post-SE, a much lower but still significant Gd-DTPA leakage was detected only in the amygdala (day 4, *p* = 0.040) and piriform (day 4, *p* = 0.016; day 10, *p* = 0.0036) and entorhinal (day 10, *p* = 0.030) cortex, proposing a recovery of BBB integrity in the other formerly affected brain regions (Fig. 1*B*). Elevated T2 values, indicative of brain edema, were present 48 h post-SE in all investigated brain regions including cerebellum (*p* < 0.045 for all brain regions), with the highest T2 value increase of 23% in the amygdala (Fig. 1*C*,*D*). At 4 d post-SE, T2 values were still increased in hippocampus (*p* = 0.035), thalamus (*p* = 0.011), and entorhinal cortex (*p* = 0.021). Subsequently, edema further decreased, and no significantly changed T2 values were present at 10 d following SE (Fig. 1*D*).

### Extent and spatiotemporal pattern of FITC-albumin extravasation following status epilepticus evaluated by histologic analyses

**Figure 2.**
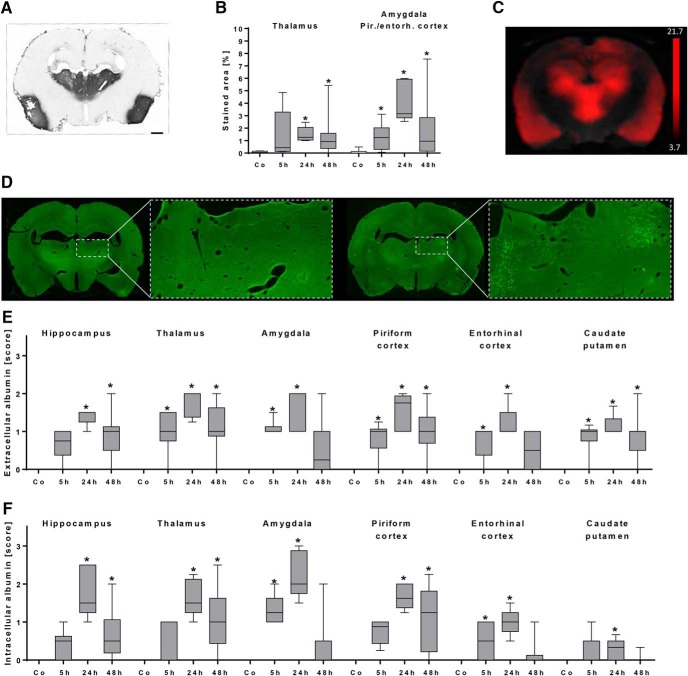
Histologic evaluation of albumin extravasation during early epileptogenesis. ***A***, Distribution of FITC-labeled albumin (FITC-Alb) after its conversion into a light microscopically visible adduct with anti-FITC-HRP and nickel-enhanced DAB in a representative section from a rat 48 h following status epilepticus (SE). The extravasation marker indicating leakage of allocated BBB is predominantly visible in the thalamus and the piriform cortex. Scale bar = 1 mm. ***B***, Quantification of DAB-positive area relative to the total section area in control rats (*n* = 10) and rats 5 h (*n* = 6), 24 h (*n* = 5), and 48 h (*n* = 16) following SE. * *p* < 0.05 compared to control, one-way ANOVA, Dunnett’s multiple comparison test. ***C***, Coronal Gd-DTPA-enhanced T1 MRI leakage map resulting from comparison between baseline and 48 h post-SE. Note the striking similarity of BBB leakage pattern in the *ex vivo* DAB-converted FITC-albumin slice (***A***) and *in vivo* contrast-enhanced MRI (***C***). Leakage *t*-map was calculated by SPM12 software (two-sample unpaired *t* test, *p* < 0.001, and a minimum cluster size of 100 voxels; scale bar displays *t*-values). ***D***, Exemplary whole-brain sections and a higher-magnified image of the thalamus from two rats, predominantly showing extracellular distribution (left, 48 h post-SE) or intracellular uptake (right, 24 h post-SE) of extravasated FITC-labeled albumin. ***E***, Semiquantitative analysis of extracellular FITC-labeled albumin in control rats (*n* = 10) and rats 5 h (*n* = 6), 24 h (*n* = 5), and 48 h (*n* = 14) following SE. ***F***, Semiquantitative analysis of intracellular FITC-labeled albumin in control rats (*n* = 10) and rats 5 h (*n* = 6), 24 h (*n* = 5), and 48 h (*n* = 14) following SE. ***E*** and ***F*** show peak values of FITC-albumin presence at 24 h post-SE. * *p* < 0.05 compared to control, Kruskal–Wallis ANOVA, Dunn’s multiple comparison test. Data are illustrated as box-and-whisker plots. Co, control, Pir./entorh., piriform/entorhinal.

Histologic analysis of DAB-converted FITC-albumin signals revealed distinct extravasation in thalamic areas, amygdala, and piriform and entorhinal cortices (Fig. 2*A*). Quantification of stained area showed significantly elevated extravasation in thalamus 24 h (*p* = 0.0017) and 48 h (*p* = 0.0028) post-SE, whereas in amygdala and piriform/entorhinal cortex, significantly increased values were found at all investigated time points (5 h, *p* = 0.038; 24 h, *p* = 0.0001; 48 h, *p* = 0.013; Fig. 2*B*). The hippocampus was moderately affected in individual animals, but signal quantification did not reach statistical significance on the group level (data not shown). Comparison of *ex vivo* extravasation pattern (Fig. 2*A*) with *in vivo* contrast-enhanced MRI leakage map revealed a very comparable spatial distribution of albumin 48 h post-SE (Fig. 2*C*). Intra- and extracellular distribution of extravasated FITC-albumin differed between individuals (Fig. 2*D*), but semiquantitative histologic group analysis of native FITC-albumin signal revealed extracellular FITC-albumin in thalamus (*p* = 0.025), amygdala (*p* = 0.0019), piriform cortex (*p* = 0.034), entorhinal cortex (*p* = 0.014), and caudate putamen (*p* = 0.0063) already 5 h following SE (Fig. 2*E*). At 24 h post-SE, extracellular FITC-albumin reached its maximum in all analyzed brain regions (*p* ≤ 0.0011), and declined again at 48 h post-SE (Fig. 2*E*). At 5 h following SE, intracellular FITC-albumin was observed only in amygdala (*p* = 0.0015) and entorhinal cortex (*p* = 0.045), whereas it was found in all analyzed brain regions (*p* ≤ 0.022) 24 h following SE (Fig. 2*F*). At 48 h following SE, it still reached significance in hippocampus (*p* = 0.0048), thalamus (*p* = 0.0017), and piriform cortex (*p* = 0.0006; Fig. 2*F*). In brain sections of control rats, neither extra- nor intracellular FITC-albumin was observed. Furthermore, FITC-albumin leakage was not visible in pilocarpine-treated rats without SE development (data not shown).

### Time course and extent of neurodegeneration following status epilepticus

**Figure 3.**
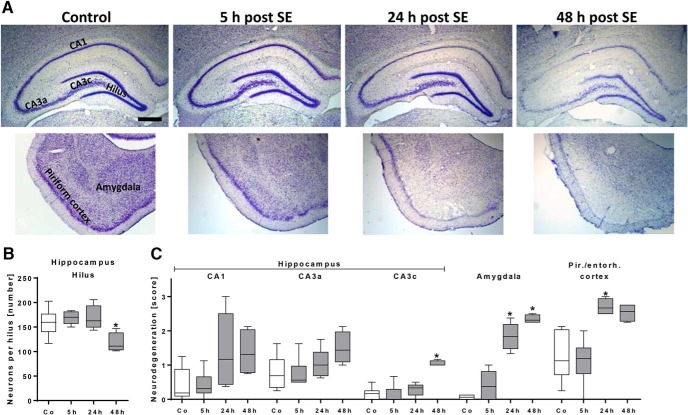
Histologic evaluation of neurodegeneration during early epileptogenesis. ***A***, Representative images of Nissl-stained forebrain sections (–3.6 mm relative to bregma; left hemisphere; hippocampus, piriform cortex/amygdala) of a control rat and rats 5, 24, and 48 h following status epilepticus (SE). Scale bar = 500 µm. ***B***, Number of hilar neurons in control rats (*n* = 6) and rats 5 h (*n* = 6), 24 h (*n* = 5), and 48 h (*n* = 4) following SE, revealing neurodegeneration only at 48 h post-SE. ***C***, Semiquantitative analysis of neurodegeneration in hippocampal subregions, amygdala, and cortical subregions in control rats (*n* = 6) and rats 5 h (*n* = 6), 24 h (*n* = 5), and 48 h (*n* = 4) following SE. * *p* < 0.05 compared to control, one-way ANOVA (***B***); Dunnett’s multiple comparison test, Kruskal–Wallis ANOVA, and Dunn’s multiple comparison test (***C***). Data are illustrated as box-and-whisker plots. CA, cornu ammonis. Co, control, Pir./entorh., piriform/entorhinal.

Semiquantitative analysis of neurodegeneration in hippocampal and cortical subregions as well as amygdala revealed neuronal loss (example shown in Fig. 3*A*) already at 24 h following SE in the amygdala (24 h, *p* = 0.018, 48 h, *p* = 0.0018) and the piriform and entorhinal cortices (24 h, *p* = 0.0097; Fig. 3*C*). At 48 h after SE, hippocampal pyramidal cells of CA3c subregion also reached statistical significance (*p* = 0.017; Fig. 3*C*). Moreover, the number of hilar mossy cells and interneurons as assessed by cell counting was significantly reduced 48 h following SE (*p* = 0.026; Fig. 3*B*).

### Colocalization of FITC-albumin with cellular or extracellular cerebral markers

**Figure 4.**
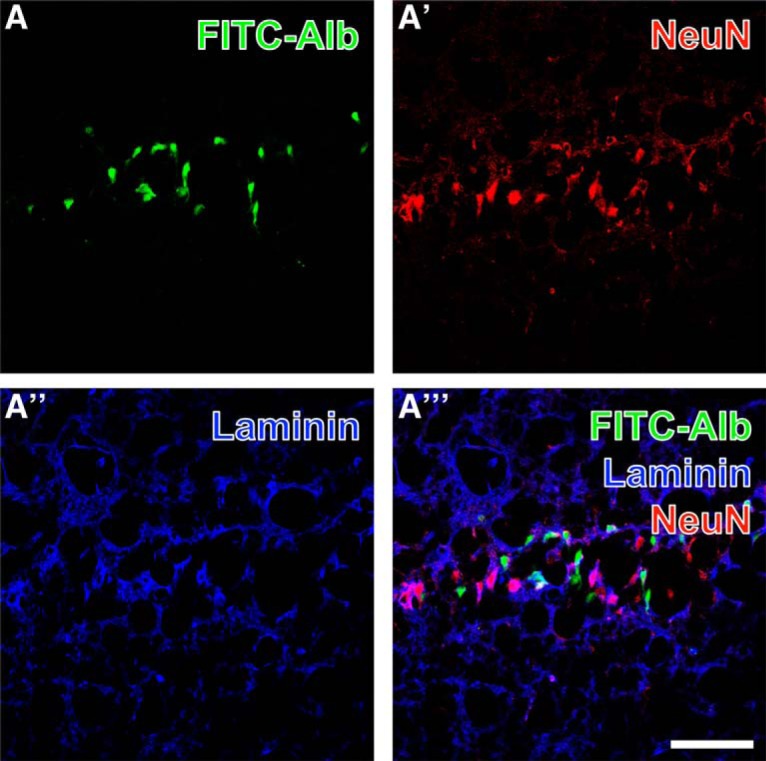
Concomitant detection of FITC-coupled albumin (FITC-Alb; ***A***) in the pyramidal layer of the hippocampal CA1 region 48 h after SE shows predominantly intracellular FITC-Alb. Neuronal somata are stained with biotinylated anti-NeuN and red fluorescent Cy3-streptavidin (***A′***), while vascular basement membranes are visualized with rabbit anti-laminin and Cy5-donkey anti-rabbit IgG (***A″***, immunosignals color-coded in blue). The overlay of staining patterns (***A″′***) elucidates only rare colocalization of FITC-Alb and NeuN-positive neurons. Scale bar = 50 µm.

Randomly selected sections from rats 24 and 48 h after SE were subjected to multiple fluorescence labeling. FITC-albumin, counterstained with biotinylated anti-NeuN (Fig. 4*A′*), displayed only minor colocalization (Fig. 4*A″′*), despite intracellular location of FITC-albumin in cells of obvious neuronal morphology (Fig. 4*A*); an example is shown for pyramidal cell layer of hippocampal CA1 region. In contrast to NeuN-positive neurons, FITC-albumin–positive cells of neuronal morphology exhibited only limited spatial overlap with endothelial basement membranes visualized by laminin immunolabeling (Fig. 4*A″*).

**Figure 5.**
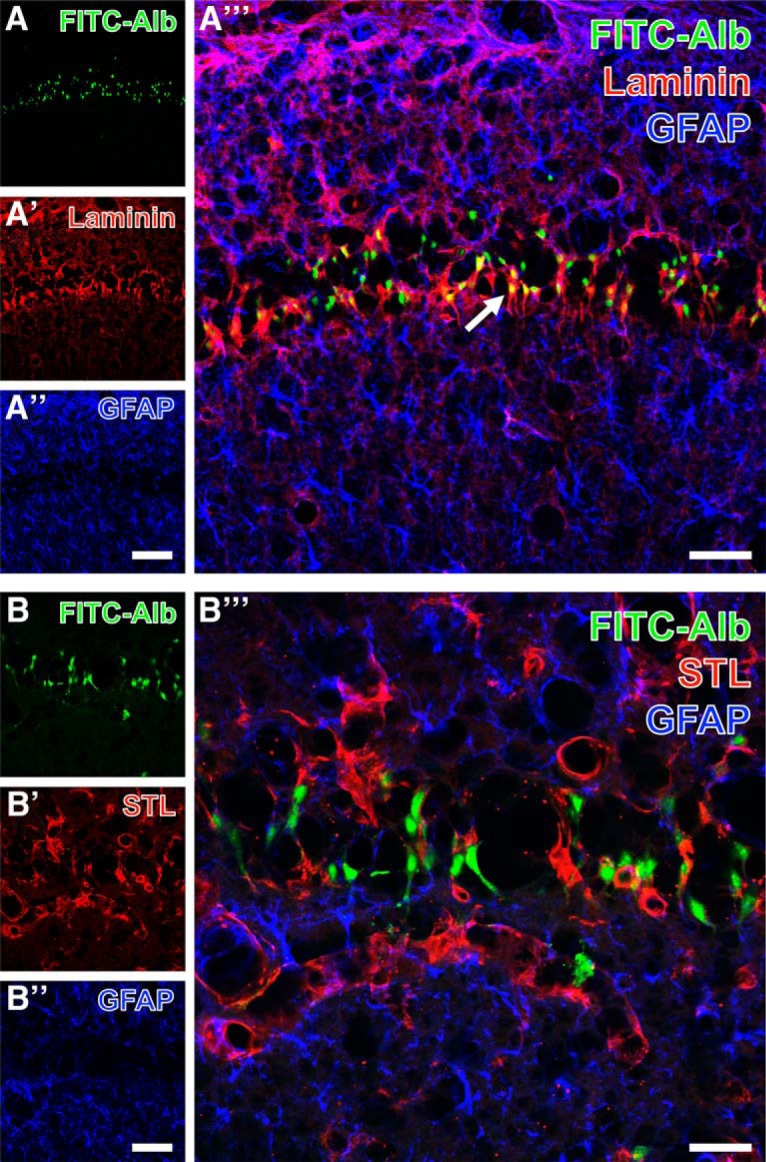
Hippocampal pyramidal cell layer in the CA1 region with FITC-coupled albumin (FITC-Alb; ***A***, ***B***) 48 h following SE and laminin-immunoreactivity in vascular basement membranes (Cy3, red; ***A′***) or endothelial binding sites for biotinylated *Solanum tuberosum* lectin (STL; Cy3, red; ***B′***), each combined with immunolabeling of astroglial GFAP (***A″***, ***B″***, Cy5, color-coded in blue) at lower (***A–A″′***) and higher (***B–B″′***) magnification. Merged staining patterns (***A″′***, ***B″′***) elucidate FITC-Alb in close vicinity to laminin-immunopositive structures (arrow in ***A″′***), but no obvious colocalization of FITC-Alb and GFAP-positive astrocytes (***A″′***, ***B″′***). Scale bars: ***A″*** (also valid for ***A***, ***A′***) = 100 µm, ***A″′*** = 50 µm, ***B″*** (also valid for ***B***, ***B′***) = 50 µm, ***A″′*** = 25 µm.

**Figure 6.**
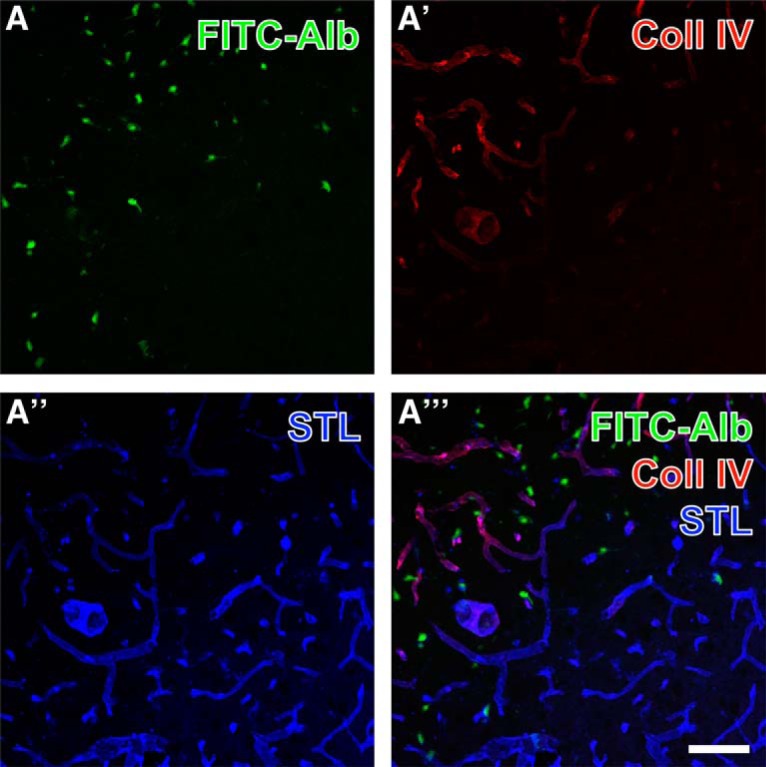
Simultaneous demonstration of thalamic FITC-coupled albumin (FITC-Alb) 24 h following SE combined with the detection of the vascular marker collagen IV and binding sites for *Solanum tuberosum* lectin (STL). FITC-Alb (***A***) is seen in neuron-like cells in SE-affected regions, mainly marked by apparently up-regulated collagen IV (Coll IV) immunoreactivity (***A′***, Cy3, red). Concomitant lectin-histochemical staining with STL (***A″***, Cy5, color-coded in blue) reveals vessels that appear thinner and of lower STL signal in tissue with detectable FITC-Alb. Vascular structures containing both Coll IV– and STL-binding sites appear purple in (***A″′***). Scale bar = 75 µm.

Spatial relationships between FITC-albumin and vascular markers combined with GFAP-expressing astroglia are shown in the hippocampal pyramidal cell layer at lower (Fig. 5*A–A″′*) and higher (Fig. 5*B–B″′*) magnification. FITC-albumin was again predominantly found in pyramidal cells, and to a lower extent in close vicinity to laminin-positive vascular structures (Fig. 5*A*), which becomes more obvious in the merged staining patterns (arrow in Fig. 5*A″′*). In parallel, GFAP-immunopositive structures (Fig. 5*A″*,*B″*) appeared separated in the overlays of staining patterns, which reveal astrocytic endfeet contacting endothelial cells stained with biotinylated STL (arrow in Fig. 5*A″′*). Thalamic FITC-albumin apparently within cells (Fig. 6*A*) was additionally counterstained by collagen IV immunolabeling of basal membranes (Fig. 6*A′*) and biotinylated STL detecting endothelial cells (Fig. 6*A″*). The even distribution of STL-stained vessels contrasted with the local upregulation of collagen IV expression. The overlay of staining patterns (Fig. 6*A″′*) revealed that FITC-albumin–positive areas concomitantly displayed up-regulated vascular collagen IV immunoreactivity that was frequently allocated with STL-binding sites also in thinned vessels.

**Figure 7.**
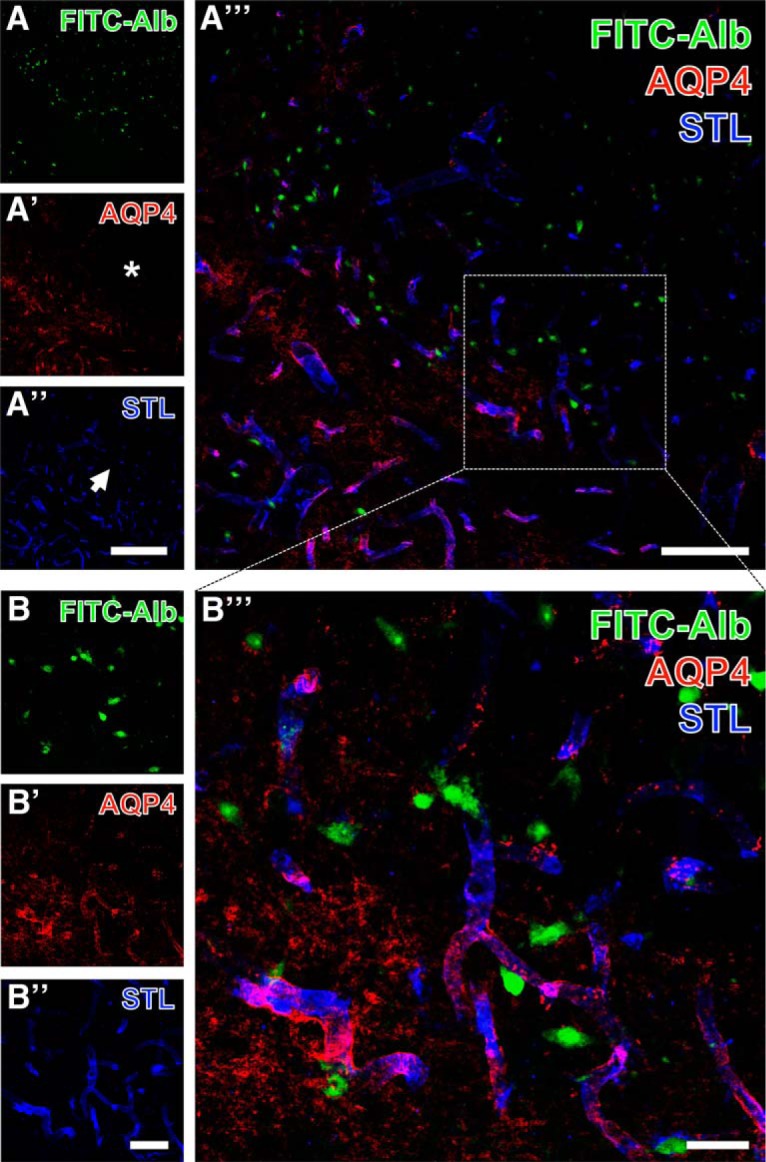
Concomitant visualization of thalamic FITC–coupled albumin (FITC-Alb) 24 h after SE with astroglial aquaporin-4 (AQP4, astrocytic endfeet) and endothelial *Solanum tuberosum* lectin (STL) binding as overview (***A–A″′***) and at higher magnification (***B–B″′***). FITC-Alb is seen in numerous neuron-like cell somata (***A***, ***B***). AQP4 immunolabeling (***A′***, ***B′***) is largely absent in FITC-Alb–positive tissue marked by asterisks in ***A′*** but distinctly expressed in adjacent areas. STL staining in the same area appears diminished (***A″***, arrow). The overlay of staining patterns (***A″′***, ***B″′***) elucidates allocated AQP4-immunoreactive astrocytic endfeet and endothelial STL-binding sites appearing as purple vessels in close vicinity to many FITC-Alb–filled cells. Scale bars: ***A″*** (also valid for ***A***, ***A′***) = 200 µm, ***A″′*** = 100 µm, ***B″*** (also valid for ***B***, ***B′***) = 50 µm, ***B″′*** = 25 µm.

Next, FITC-albumin and STL staining were combined with the immunodetection of AQP4 known as marker for astrocytic endfeet. An example of this triple staining is shown in the thalamus at lower (Fig. 7*A–A″′*) and higher (Fig. 7*B–B″′*) magnification. SE-affected regions as indicated by intracellular FITC-albumin (Fig. 7*A*,*B*) were largely devoid of immunosignals for AQP4 (asterisks in Fig. 7*A′*,*B′*) whereas adjacent tissue within the affected areas displayed much higher levels of vessel-associated AQP4. In parallel, STL staining in the affected regions either was diminished (Fig. 7*A″*, arrow) or appeared unaltered (Fig. 7*B″* ,*B″′*).

**Figure 8.**
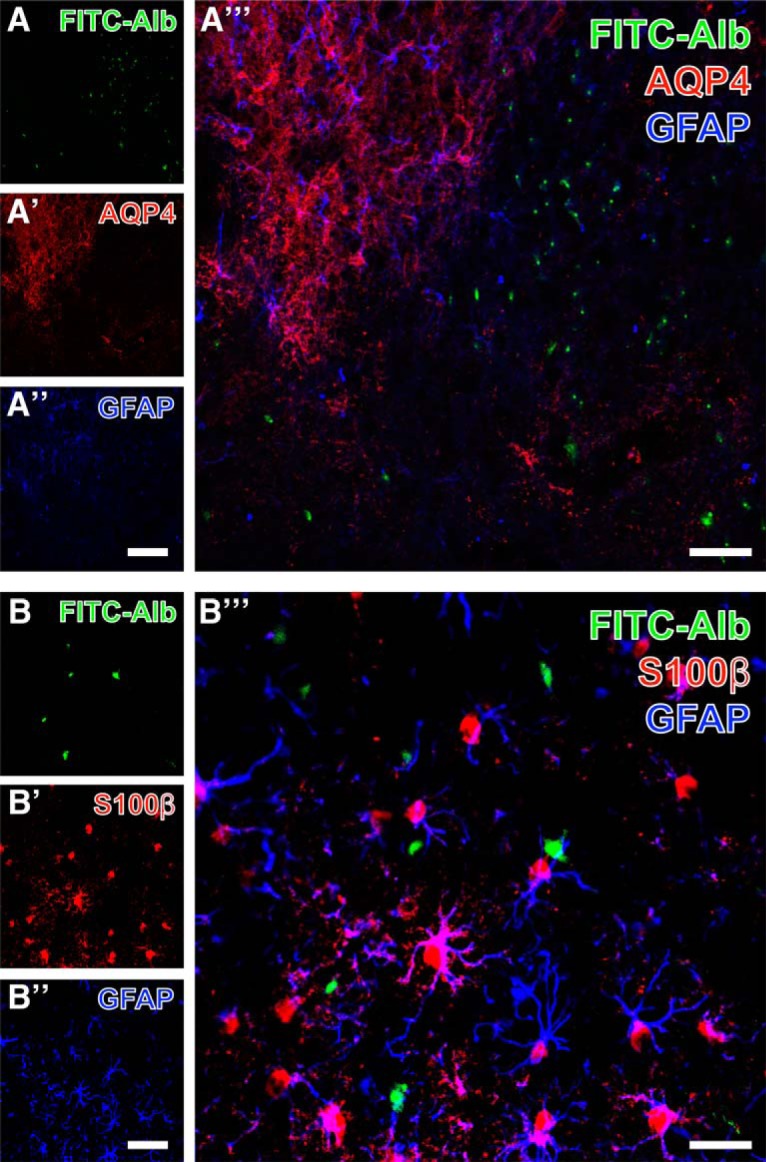
Detection of GFAP and cellular FITC-coupled albumin (FITC-Alb) in SE-affected thalamus 48 h after SE onset combined with immunolabeling of aquaporin-4 (AQP4; ***A–A″′***) or S100β (***B–B″′***). FITC-Alb in ***A*** is seen in region devoid of Cy3 staining for AQP4 (***A′***) and diminished GFAP immunosignals (***A″***). The overlay of staining patterns (***A″′***) reveals a nearly complementary occurrence of FITC-Alb and AQP4 expression, but also shows remnants of mostly punctuate GFAP-immunoreactive structures. At higher magnification, in another thalamic area from the same animal, Cy3 counterstaining of S100β (***B′***) predominantly reveals astroglial somata with numerous processes, whereas the immunodetection of GFAP with Cy5 (***B″***, color-coded in blue) visualizes astrocytic intermediate filaments. The merge of staining patterns (***B″′***) clearly demonstrates numerous astrocytes coexpressing both astroglia-specific markers but lacking allocated FITC-Alb. Scale bars: ***A″*** (also valid for ***A***, ***A′***) = 100 µm, ***A″′*** = 50 µm, ***B″*** (also valid for ***B***, ***A′***) = 50 µm, ***B″′*** = 25 µm.

To analyze the spatial relationships between astroglial markers as well as between astrocytes and FITC-albumin deposition, the immunolabeling of AQP4 was combined with the detection of GFAP (Fig. 8*A–A″′*) or S100β (Fig. 8*B–B″′*) in SE-affected thalamic tissue containing FITC-albumin–filled cells. FITC-albumin (Fig. 8A) was observed in regions lacking apparent AQP4 immunoreactivity (Fig. 8*A′*) and with weakened GFAP immunodecoration (Fig. 8*A″*). Merged staining patterns (Fig. 8*A″′*) indicated a nearly complementary occurrence of FITC-albumin and AQP4 expression together with remnants of mostly punctate GFAP-immunoreactive structures. Subsequently, FITC-albumin–positive areas Fig. 8B) were counterstained with anti-S100β (Fig. 8*A′*), which predominantly reveals astroglial somata with numerous processes, whereas the immunodetection of GFAP with Cy5 (Fig. 8*A″*, color-coded in blue) visualizes astrocytic intermediate filaments. The merged staining patterns (Fig. 8*A″′*) clearly demonstrated numerous astrocytes coexpressing both astroglia-specific markers but lacking allocated FITC-albumin.

**Figure 9.**
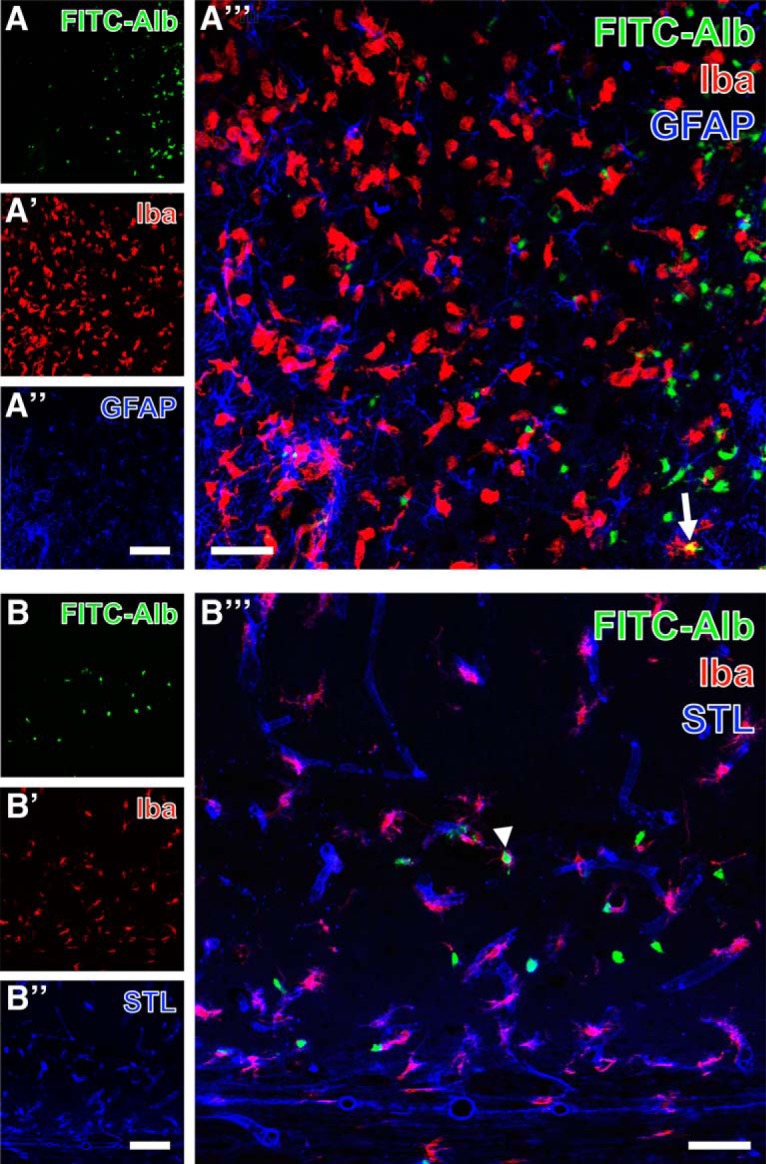
Demonstration of FITC-coupled albumin (FITC-Alb) and Iba-immunoreactive microglia/macrophages combined with thalamic GFAP immunolabeling 48 h after SE onset (***A–A″′***) and the detection of hippocampal *Solanum tuberosum* lectin (STL)-binding sites 24 h after SE onset (endothelial cells, ***B–B″′***). FITC-Alb in the thalamus is predominantly visible within neuron-like cells (***A***). The same region contains Iba-immunopositive ameboid microglia that are even more strongly labeled in adjacent tissue with less FITC-Alb (***A′***). In parallel, proliferating astrocytes are revealed by GFAP immunostaining (***A″***). The merge of staining patterns (***A″′***) allows for the identification of single FITC-Alb–filled immune cells displaying the mixed color yellow (arrow in ***A″′***). Additionally, hippocampal FITC-Alb–positive cells are allocated with Cy5 staining of STL-binding sites and Cy3-immunolabeling of Iba (arrowhead, ***B″′***). Scale bars: ***A″***, ***A″*** (also valid for ***A***, ***A′***, ***B***, ***B′***) = 100 µm, ***A″′***, ***B″′*** = 50 µm.

For the simultaneous detection of astroglia and microglia/macrophages, FITC-albumin was counterstained with GFAP and Iba, an example of which is shown for the thalamus (Fig. 9*A–A″′*). SE-induced leakage of the BBB again led to extravasation of FITC-albumin followed by its internalization by neuron-like cells (Fig. 9*A*). Numerous Iba-immunopositive ameboid microglial cells were seen in the same tissue (Fig. 9*A′*) and were intermingled with active, proliferating astrocytes (Fig. 9*A″*). The overlay of staining patterns (Fig. 9*A″′*) only rarely indicated FITC-albumin–filled glial cells (Fig. 9*A″′*). Cy3 immunodetection of Iba (Fig. 9*B′*) was combined with the Cy5 staining of STL-binding sites that were found in vessels as well as in ameboid perivascular microglia/macrophages (Fig. 9*B″*). A majority of perivascular Iba-positive activated microglia displayed in the overlay of staining patterns (Fig. 9*B″′*) both lectin and immunohistochemical labeling. FITC-albumin was occasionally also found in Iba-positive microglia (arrowhead).

**Figure 10.**
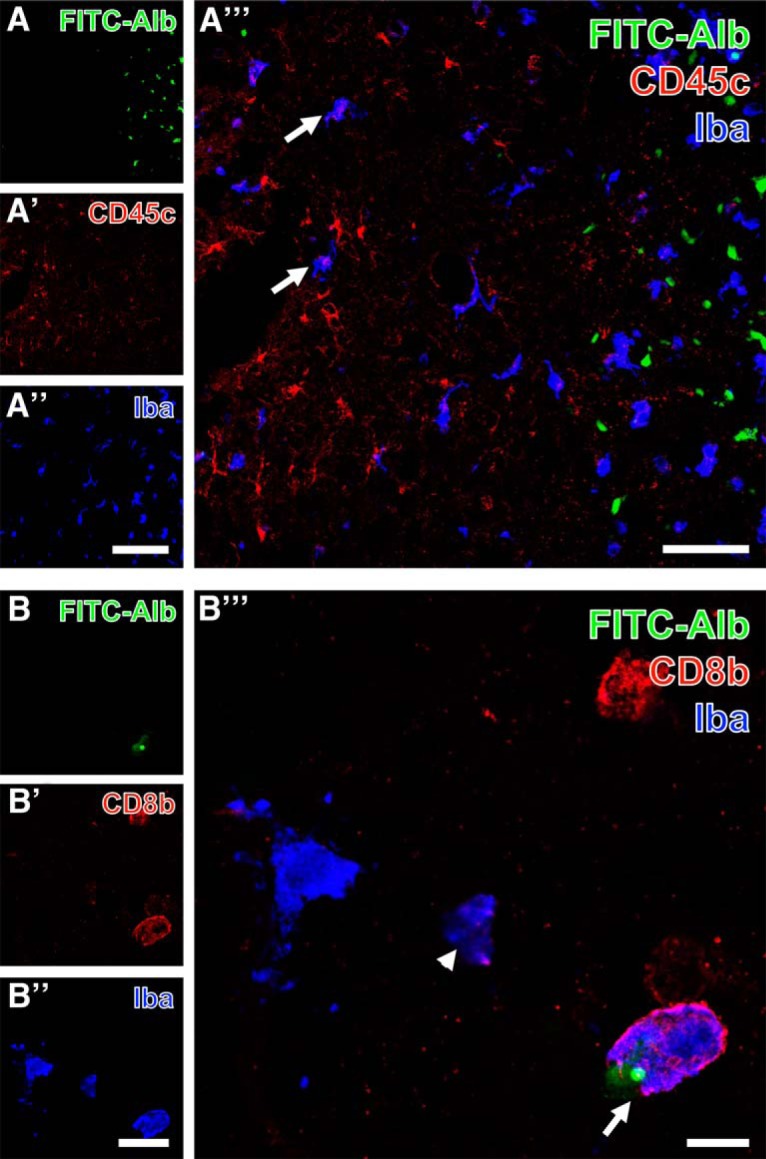
Triple fluorescence labeling of FITC-coupled albumin (FITC-Alb) and Iba combined with the immunodetection of immune cells expressing either CD45c (***A–A″′***) or CD8b (***B–B″′***) in thalamic regions 48 h after SE. FITC-Alb–filled cells are here restricted to a clearly delineated zone (***A***), whereas CD45c-immunodetection (***A′***) visualizing the leukocyte common antigen is stronger in tissue devoid visible FITC-Alb and Iba (***A″***), which is seen in more evenly distributed ameboid immune cells. The overlay of staining patterns (***A″′***) clearly shows several cells coexpressing both microglial markers (exemplified by arrows in ***A″′***), whereas all FITC-Alb–stained cells appear monolabeled. At higher magnification, one cell displays not only a cytoplasmic label with FITC-Alb (***B***), but also a small, round compartment with a much stronger fluorescence signal. This cell and three other cells with similar ameboid appearance are additionally Cy3-labeled for CD8b indicating a subset of lymphocytes, whereas Cy5 immunostaining of Iba (***B″***, color-coded in blue) is also present in apparently CD8b-immunonegative cells. The overlay of staining patterns clearly demonstrates two cells coexpressing both immune cell markers either containing FITC-Alb (arrow) or being devoid of FITC-Alb (arrowhead). Scale bars: ***A″*** (also valid for ***A***, ***A′***) = 100 µm, ***A″′*** = 50 µm, ***A″*** (also valid for ***B***, ***A′***) = 25 µm, ***B″′*** = 10 µm.

For specifying immune cells exemplified in the thalamus 48 h after SE, Iba was counterstained with either anti-CD45c in activated microglia (Fig. 10*A–A″′*) or anti-CD8b in T lymphocytes (Fig. 10*B–B″′*). FITC-albumin–filled cells (Fig. 10*A*) were observed in a clearly delineated zone with reduced Cy3 immunolabeling of CD45c (Fig. 10*A′*), while Iba immunoreactivity (Fig. 10*A″*) was found in ameboid immune cells accumulating close to FITC-albumin–positive cells. Merged staining patterns (Fig. 10*A″′*) elucidated several cells coexpressing both microglial markers (arrow in Fig. 10*A″′*). At higher magnification, FITC-albumin (Fig. 10*B*) was occasionally found in immune cells expressing CD8b (Fig. 10*B′*) as well as Iba (Fig. 10*B″*), which is exemplified in the overlay (Fig. 10*B″′*) by the arrow-marked cell.

Table 2 provides a survey of the results gained by analysis of fluorescence staining. In brain sections from control rats, neither extra- nor intracellular FITC-albumin was observed. Further, none of the alterations in immunosignals or abnormal staining patterns described above were seen in sections from control rats that underwent the identical staining procedures (not shown).

**Table 2.** Summary of results gained by analysis of fluorescence staining after SE

Marker	Target	Colocalization with FITC-albumin?	Altered immunosignal in FITC-albumin-positive vs. negative areas?
NeuN	Neurons	(Yes)*	n.d.
GFAP	Astroglia	No	Reduced
Iba1	Microglia	Yes	n.d.
STL	Vasculature/endothelial cells, perivascular microglia	No	Reduced
Laminin	Vascular basement membranes of endothelium	Yes	No
Collagen IV	Vascular basement membranes of endothelium	No	Elevated
AQP4	Astroglial endfeet	No	Reduced
S100β	Astroglial somata	No	No
CD45c	Activated microglia	No	Reduced
CD8b	Subset of T cells	Yes	No

AQP4, aquaporin-4; GFAP, glial fibrillary acidic protein; Iba1, ionized calcium binding adaptor molecule 1; n.d., not determined; NeuN, neuronal nuclei; STL, *Solanum tuberosum* lectin.

*But predominantly in NeuN-immunonegative cells displaying neuronal morphology.

## Discussion

SE and prolonged febrile seizures represent clear risk factors for developing temporal lobe epilepsy, which is strongly supported by data from human and animal studies (French et al., 1993; Mathern et al., 1995; Scott et al., 2003; Patterson et al., 2014), and BBB leakage is proposed to represent a crucial event contributing to epileptogenesis (Marchi et al., 2007b; Friedman et al., 2009). The purpose of this study was to provide comprehensive data on the spatiotemporal evolution of SE-induced BBB leakage *in vivo* by translational MR imaging and *ex vivo* by complementary immunohistochemical analyses characterizing the response of NVU components to the epileptogenic brain insult. Our data provide important information for therapeutic intervention after epileptogenic brain insults and suggest that a very prompt BBB-stabilizing intervention will be necessary to prevent distinct albumin extravasation and subsequent pro-epileptogenic consequences following SE. The main findings are as follows: (i) SE-induced BBB leakage peaks between 1 and 2 d post-SE, affecting main epileptogenesis-associated brain regions, and rapidly declines thereafter; (ii) increase in T2-weighted MRI mainly follows the time course of contrast agent extravasation; (iii) at the time of maximum BBB leakage, extravasated albumin colocalizes with the perivascular basement membranes, neurons, and brain immune cells, but not with astrocytes; (iv) albumin-positive areas are characterized by reduced immunoreactivity for astroglial markers (GFAP, AQP4), as well as endothelial STL-binding sites, whereas collagen IV, a marker of perivascular basement membranes, appears elevated.

Uncovering the time course of BBB leakage is of particular relevance for timing therapeutic intervention during epileptogenesis. Although the temporal evolution of increased BBB permeability was lately determined for another post-SE model of epileptogenesis (van Vliet et al., 2016), respective data for the pilocarpine model, which is often applied to examine therapeutic intervention during epileptogenesis (Löscher, 2012), were not available so far. Here, we applied a translational MRI-based imaging method, which we recently identified to be the method of choice for detection and quantification of BBB leakage (Breuer et al., 2017). The leakage displayed its maximum about 2 d after SE and clearly declined on day 4, suggesting a partial recovery of BBB integrity in formerly affected brain regions such as hippocampus and thalamus (Fig. 1). The persistently increased BBB permeability in the piriform cortex at day 10 might indeed be indicative of continuous epileptogenesis in this animal model. Comparable results from other post-SE models support this idea. After electrically induced SE, van Vliet et al. (2007) found increased albumin extravasation, histologically assessed by analyses of fluorescein signals, still in the latent and, to a minor extent, also in the chronic epilepsy phase in several epileptogenesis-associated brain regions including the piriform cortex. The same group reported persistent leakage of contrast agent at 3 and 6 wk after kainate-induced SE as assessed by T1-weighted MRI in the amygdala/piriform cortex (van Vliet et al., 2016). After paraoxon-induced SE, Bar-Klein et al. (2017) observed BBB leakage in the piriform network at 2 d and 1 mo, but not at 1 wk, post-SE in rats identified as epileptic later on, nonetheless suggesting a persistent affection of this region after SE. Interestingly, van Vliet et al. (2016) found a correlation between BBB leakage in the piriform cortex and seizure frequency in the chronic phase, and Bar-Klein et al. (2017) identified BBB leakage in the piriform network as a predictive marker for epilepsy development underlining the importance of investigating changes accompanying albumin extravasation on a cellular level.

Additionally, we evaluated T2-weighted MRI to spatio-temporally assess SE-induced brain edema and inflammation, as T2-weighted MRI was suggested also as an indicator of active inflammation (Michoux et al., 2015; Peixoto-Santos et al., 2017). T2-signal increase was found to a lesser extent than T1-signal increase, but with a similar spatiotemporal profile (Fig. 1), suggesting cerebral edema to appear predominantly early after SE and to resolve soon thereafter. Our findings correspond to those of previous preclinical studies by Roch et al. (2002), Choy et al. (2010), and Duffy et al. (2014), who detected peaks of T2 intensity around 2–4 d following pilocarpine-induced SE. Importantly, our data are also in line with clinical data on hippocampal edema to occur within 48 h after SE in human subjects (VanLandingham et al., 1998; Scott et al., 2002). In a recent study, we longitudinally assessed the time course of microglia activation after pilocarpine-induced SE by [^11^C]PK11195 positron emission tomography (Brackhan et al., 2016). We found inflammation peaking between 1 and 2 wk post-SE, i.e., distinctly later than T2-signal increase observed in the present study, suggesting that T2-weighted MRI reflects not only microglia activation but also other aspects of post-SE neuroinflammation. As the T2 MRI signal is strongly dependent on stationary water concentration, brain water homeostasis might be impaired by altered astrocytic AQP4 function. A recent study in human TLE patients indeed demonstrated that increased T2 relaxation time correlates with astrogliosis, microgliosis, and chondroitin sulfate proteoglycan expression (Peixoto-Santos et al., 2017). In the same patients, expression of AQP4 in astrocytic endfeet was distinctly reduced.

To substantiate our analysis, we performed additional histologic assessment of FITC-albumin extravasation. Importantly, extravasation patterns analyzed *ex vivo* were generally congruent with *in vivo* MRI findings (Fig. 1 and 2). Our results corroborate peak albumin extravasation between 24 and 48 h after SE. While converting the albumin signal into a light-microscopically visible adduct (Michalski et al., 2010) provides quantitative values even at electron microscopic level (Krueger et al., 2015), the score-based analysis gives similar results and allows the differentiation of extra- and intracellular appearance of green fluorescence. Detection of intracellular FITC-albumin at 5 h post-SE demonstrates that cells of neuron-like shape are pathologically affected at this time point to allow albumin uptake. Here, we used intravenous injection of FITC-albumin with the intention to reveal the actual extent and localization of extravasated albumin at the chosen time points after SE. In contrast, staining of endogenous albumin at the respective time points would have resulted in information about the cumulative amount of albumin extravasated during the whole time span between SE and time of sacrifice. There is no evidence that dyes of low molecular weight (compared with the molecular weight of albumin), covalently bound or not, alter the crossing properties of albumin at the BBB. Furthermore, crossing of endogenous albumin will be relevantly influenced by exogenous FITC-albumin only when given in high amounts. Notably, the administered amount of FITC-albumin (100 mg/kg) relates to <5% of the average endogenous albumin blood concentration in adult Sprague-Dawley rats (3 g/dl, Zaias et al., 2009), resulting in a slight underestimation, if any, of the amount of extravasated albumin.

Histologic examinations were performed to evaluate consequences of albumin extravasation for the NVU in further detail (Table 2). Areas with albumin extravasation and uptake in neuron-like cells also showed loss of NeuN immunoreactivity and colocalization with the vascular basement membrane marker laminin (Fig. 3). It was described before that neurons can lose NeuN immunoreactivity despite preservation of nuclear membrane integrity after an ischemic brain insult (Ünal-Cevik et al., 2004). Ünal-Cevik and colleagues suggested that this loss of NeuN antigenicity might be caused by severe insult-induced metabolic perturbations of neurons or by depletion or alteration of the antigen. Therefore, the colocalization of FITC-albumin with NeuN-negative cells of neuronal morphology, as described here, indicates FITC-albumin uptake by damaged, but morphologically non-affected neurons. This assumption is supported by data on extravasated albumin-bound Evans Blue after SE often in Fluoro-Jade B–positive, i.e., dying, neurons (van Vliet et al., 2007). This is also in line with the distinct neurodegeneration found on days 1 and 2 after SE in Nissl-stained temporal lobe subregions (Fig. 2). Unfortunately, Fluoro-Jade staining representing the best evaluated marker of dying neurons could not be evaluated here, as its light emission profile and that of fluorescein are considerably interfering. In concordance with our data, neurons were reported to be the only or the major cell type containing albumin following acute seizures or SE, i.e., in an affected brain (Marchi et al., 2007a; van Vliet et al., 2007, Frigerio et al., 2012). Accordingly, neuronal uptake was frequently observed in regions of Evans Blue extravasation in the porcine hippocampus following osmotic BBB impairment and adherent acute seizures (Marchi et al., 2007b). However, *in vitro* exposure of naive rat cortical brain slices to FITC-albumin or prolonged (72-h) infusion of albumin into the lateral ventricle of naive mice did not result in colocalization of albumin with neurons (Ivens et al., 2007; Weissberg et al., 2015). These conflicting findings suggest that albumin behaves differently in the seizing versus the naive brain, and that albumin uptake into neurons might be favored by an impaired environment. This assumption is also supported by Michalak et al. (2012), who tracked the uptake of extravasated IgG into neurons in chronic TLE patients and rats during epileptogenesis.

Assessment of albumin colocalization with a variety of further cellular markers also exposed spatial overlap of albumin with microglia and CD8b-positive T cells (Figs. 3–9). Surprisingly, and despite the use of three markers labeling different astrocytic compartments—i.e., intermediate filaments (GFAP), predominantly somata (S100β), and endfeet (AQP4)—astroglia were devoid of detectable FITC-albumin in the present study. This is in contrast to the reported selective transport of albumin into astrocytes *in vitro* in cortical brain slices or cultured astrocytes (Ivens et al., 2007; Bar-Klein et al., 2014), but in general accordance with observations after electrically induced SE (van Vliet et al., 2007) and after acute seizures (Marchi et al., 2007a). Furthermore, we show that in FITC-albumin–positive areas after SE, staining intensity of astrocytic markers was reduced (GFAP) or lacking (AQP4), indicating a severe disturbance of the endothelial-neuronal coupling mediated by astrocytes. In earlier studies, we and others observed significantly increased activation of astroglia at the light-microscopic level in epileptogenesis-associated brain regions after pilocarpine-induced SE (cf. Zhang et al., 2015; Brackhan et al., 2016). Applying laser-scanning microscopy, we here observed the reduced immunoreactivities for GFAP and AQP4 in albumin-positive areas in proximity to the blood vessels of the NVU, i.e., this finding holds mainly for the astrocyte compartments contributing to BBB constitution. This may be due to a locally higher albumin concentrations or more distinct alterations in ion concentrations close to leaky brain capillaries. Reduced AQP4 staining was also described by Lee et al. (2012a) in mice during epileptogenesis after kainate-induced SE. Importantly, partial loss of perivascular AQP4 was also found in hippocampi of epilepsy patients (Eid et al., 2005). Very recently, Peixoto-Santos et al. (2017) reported decreased AQP4 polarity, i.e., loss of AQP4 expression in astrocyte proximal processes and endfeet close to brain blood vessels of TLE patients. Additionally, our observations suggest that at sites of albumin leakage, components of STL-stained endothelial cells are less present than in areas without visible FITC-albumin. In combination with the reduced AQP4 and increased collagen IV immunoreactivity, these alterations point to a distinct adaptive reorganization process of the NVU vasculature taking place within 48 h after SE. The obvious impairment of astrocytes (reduced GFAP and AQP4) might entail disordered or dying neurons, e.g., by reduced glutamate uptake from the extracellular space resulting in neurotoxic concentrations, or by impaired astrocytic energy supply for neurons, an assumption supported by *in vivo* PET imaging studies demonstrating cerebral glucose hypometabolism shortly after SE (Goffin et al., 2009; Guo et al., 2009; Jupp et al., 2012; Lee et al., 2012b; Zhang et al., 2015).

As collagen IV is a stabilizing constituent in the vascular architecture of the NVU and part of the immunologic BBB (Dyrna et al., 2013), the observed increase in collagen IV immunoreactivity at sides of BBB leakage could represent the beginning of local repair processes to re-establish BBB integrity. Interestingly, increased collagen IV expression was also observed following other brain insults leading to NVU/BBB injury such as experimental stroke (Hawkes et al., 2013). Moreover, colocalization of FITC-albumin with brain immune cell markers (Iba, CD45c for activated microglia) and CD8b (for T cells) did not reveal any obvious cellular preference, suggesting involvement of the entire brain immune system in epileptogenesis-associated remodeling of the NVU.

In conclusion, we provide a detailed *in vivo* and *ex vivo* analysis of the spatiotemporal course of BBB leakage and associated NVU alterations, focusing on the early time period post-SE in a widely used rat model of epileptogenesis. Our data suggest that BBB damage is an important factor triggering epileptogenesis-associated changes and arises very soon after SE. Subsequent degenerative events at the level of the NVU, including degeneration of brain vessels, astrocytes, and neurons, seem to outweigh reconstructive processes. The seizing brain with leaky BBB seems to promote predominantly neuronal albumin uptake, an observation requiring further investigation to define its role in epilepsy development. Taken together, our data support the suggestion that early BBB-restoring treatment, such as isoflurane (Bar-Klein et al., 2016), glucocorticoids (Marchi et al., 2012), or levetiracetam (Itoh et al., 2016), might be one reasonable component of rational therapeutic intervention to ameliorate the development of temporal lobe epilepsy and other detrimental sequelae of SE.

## Synthesis

Reviewing Editor: Maiken Nedergaard, University of Rochester Medical Center Decisions are customarily a result of the Reviewing Editor and the peer reviewers coming together and discussing their recommendations until a consensus is reached. When revisions are invited, a fact-based synthesis statement explaining their decision and outlining what is needed to prepare a revision will be listed below. Note: If this manuscript was transferred from JNeurosci and a decision was made to accept the manuscript without peer review, a brief statement to this effect will instead be what is listed below.

This is a solid and important study mapping in great details the effect of pilocarpine induced seisures on the BBB and reactive changes i glia. The authors have defended teh critique well in the rebuttal letter. I see no reason for re-review
